# Cadet Corps as Health Centres

**Published:** 1923-03

**Authors:** R. J. E. Hanson

**Affiliations:** Surgeon Captain R.N.V.R., Hon. Sec. of the Imperial Cadet Association. 71 Pall Mall, S.W.1


### Cadet Corps as Health Centres.

SIR,—The magic wand of a statesman, long overdue, is now needed to give effect to your suggested "Cadet Corps as Health Centres." The stirps of our race is grand. How deplorable, then, if the evil influence of the Great War on the stamina and moral of our 1905-8 age groups (1,600,000 lads) be unredressed a moment longer! In the later age groups, rickets defect prevails in very many children in both urban and rural areas. Preventive medicine will never come into its own, nor Public Health be surely founded, until we attain to a carefully planned routine for the guidance and kindly control of all our youths.

Our profession, an exacting and dangerous calling, possesses the confidence and affection of the nation. It is then surely our duty to advise and to secure a universal Cadet System in the Mother Country, not forgetting the Irish boys across the sea. Australasia has provided discipline, hygienic clothing, physical training, games and 10 days' annual holiday camp for all its boys. We should do the same. Here, Lord Haig's training area organisation is ready to hand, an admirable skeleton only awaiting its complement of lads to bring health and happiness and graded proficiency to all. Here, overcrowding is the main fault in the environment of the youngsters. Here, I can state, as an owner of working-class property, that no scheme of housing can ever be economical or satisfactory that does not embrace careful management, on the lines laid down by Octavia Hill. We can show the boys what a healthy outlook and environment should be, and as "Passed Cadets" they will attain thereto, and home amenities and decencies will be preserved.

It is of interest to note that the popular Australasian Cadet System was due to the initiative and organising ability of a doctor and a schoolmaster. In the Union of South Africa there is also universal grip of their boys and adolescents. In the Dominion of Canada evolution is on the same plan. Where are we? Last, but not least, I am sure that the profession will lend our boys a hand.

R. J. E. HANSON,
Surgeon Captain R.N.V.R.,
Hon. Sec. of the Imperial
Cadet Association.

71 Pall Mall, S.W.1.

